# Are We Where We Want to Be in Undergraduate Pathology Education?

**DOI:** 10.5146/tjpath.2023.13048

**Published:** 2024-05-18

**Authors:** Sibel Sensu, Nusret Erdogan

**Affiliations:** Department of Pathology, İstinye University Faculty of Medicine, Istanbul, Turkey

**Keywords:** Undergraduate, Pathology education, Medical education

## Abstract

**Objective: **This review which aims to examine the recent and current status of pathology education in medical schools, and covers the publications related to undergraduate pathology education published between 2010 January and June 2023.

**Material and method: **A search was performed through PubMed, Google Scholar, Semantic Scholar, and Ulakbim search engines for the Science Citation Index, Science Citation Index Expanded, Emerging Sources Citation Index, Directory of Open Access Journals, Scopus, PubMed as well as TR Dizin indexed articles. The findings are categorized into two periods as 2010 January - 2020 April (pre-COVID-19 pandemic) and May 2020 - 2023 June. A total of 24 reviews/editorials/letters to the editor and 63 research articles in the pre-pandemic period and 11 reviews/editorials/ letters to the editor and 35 research articles between 2020 May and 2023 June are included in the analysis.

**Results: **Currently, medical education generally depends on core education programs with defined learning objectives and outcomes. Moreover, problem-based, case-based, and team-based interactive learning are being used along with traditional didactic courses. Additionally, digital/web-based/remote education methods have gained prominence after the COVID-19 pandemic. The virtual or augmented reality and 3D drawing applications are offered as a solution for the autopsy and macroscopy courses. A scarce number of publications are found on measuring and evaluating the effectiveness of learning.

**Conclusion: **Artificial intelligence in pathology education is a topic that looks likely to become important in the near future. National and international comprehensive standardization is a necessity. A joint effort and collective intelligence are needed to achieve the desired goals in undergraduate pathology education.

## INTRODUCTION

In order to protect and sustain public health, the doctors of the modern age must be equipped with not only more knowledge compared to the past but also with different skills. In today’s world, where digitization has permeated every aspect of life, doctors must be experts who can easily access unlimited sources of information and find the most accurate answers to their questions in the most convenient manner within this vast sea of knowledge. Questioning, analyzing, synthesizing, integrating information, and adopting solution-oriented approaches are essential within this process. The doctors of the future must be able to act quickly, think fast, and make rapid decisions. In addition, those who choose the medical profession must be individuals who value humanity, are compassionate, reliable, respectful to nature, aware of the responsibility of their work, capable of leadership, inclined towards teamwork, curious, eager to learn, open to development, and resilient at work (1,2)

Since the 1950s, new approaches have been developed to train the doctors of the future by creating core education programs with defined learning objectives and outcomes. Traditional lectures where each discipline was explained in detail have been replaced by integrated education, which includes clustered courses organized around systems (3,4). Methods such as problem-based, case-based, and team-based learning have started to take place in the educational life. Today, efforts are being made to move to interactive education models where students are more active and access information through research. New educational methods are combined with the old, didactic style of education to create up-to-date curricula. It is believed that with this system, it will be possible to train the doctors of the modern age (4–6).

### What do we Expect from Medical Students in Pathology Courses?

In the new medical school curriculum, the question of “How much pathology should be taught to medical students?” is a topic of discussion. According to the current approach, the competencies that medical students should gain in pathology courses are as follows: they should learn the causes, basic mechanisms, and processes (etiopathogenesis) of diseases, integrate disease mechanisms into organ system pathologies, and be able to adapt this knowledge to the clinic for diagnosis and treatment. While preparing the curriculum, it is necessary to determine the learning objectives and optional sub-objectives for each competency as well as to measure and evaluate the level of learning, integration, and use of knowledge (4,7). In addition, it is aimed that the graduates know and understand the role of the pathologist, the importance of histopathology in patient diagnosis, the examinations that will be required according to clinical conditions, the limits of histopathology and cytopathology, and the method of sending biopsy or cytology samples to the pathology laboratory (8) (Table 1).

**Table 1:** Main objectives of pathology education at medical schools.

Students should;
Learn the causes, basic mechanisms, and processes of diseases.
Integrate disease mechanisms into organ system pathologies.
Apply this knowledge to the clinic for diagnosis and treatment.
Understand the role of the pathologist and the importance of histopathology in patient diagnosis.
Know which examinations will be necessary according to clinical situations and be aware of the limits of histopathology
Learn how to send biopsy or cytology samples to the pathology laboratory.

The pathology curriculum has to be classified as need-to-know, want-to-know and good-to-know and, it should be integrated with clinical sciences. Furthermore, problem-based learning, case-based learning, and other interactive teaching methods should be implemented along with didactic lectures. Finally, evaluation methods should be arranged to give less emphasis on morphology and more weight on clinical interpretations (1–7).

## OBJECTIVES, METHODS and SAMPLE CHARACTERISTICS

The contribution of pathology curricula and educational methods in medical schools to the training of contemporary doctors in our country, as well as worldwide, is a subject of debate. In this review, publications related to pathology education in medical schools were examined by searching through PubMed, Google Scholar, Semantic Scholar, and Ulakbim search engines between 2010 January and 2023 June. The keywords, “undergraduate medical pathology education” for articles in English and “mezuniyet öncesi tıp patoloji eğitimi” for the ones in Turkish were used during the literature search. Publications indexed in Science Citation Index (SCI), Science Citation Index Expanded (SCIE), Emerging Sources Citation Index (ESCI), Directory of Open Access Journals (DOAJ), Scopus, PubMed, as well as TR Dizin (National Citation Index) were included in the study, while publications not indexed in these databases were excluded. The “PRISMA 2020 – Systematic Review Checklist” was utilized at every stage of the study.

**Figure 1:**
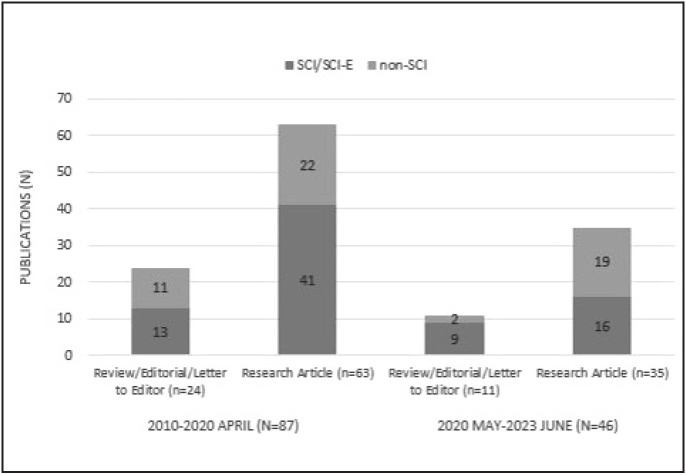
Distribution of publications between 2010-2023 June (n=133).

During the literature search, the impact of the COVID-19 pandemic, which affected the entire world since the beginning of 2020, was also observed in the field of pathology education. Therefore, in this study, it was considered to analyze the publications before April 2020, separately. The search findings were categorized into two periods as 2010 January - 2020 April and 2020 May - 2023 June. During the period from 2010 to 2020 April, a total of 24 reviews/editorials/letter to the editor and 63 research articles were eligible for the analysis (n=87). From 2020 May to 2023 June, 11 reviews/editorials/ letter to the editor and 35 research articles were included in the analysis (n=46). The distribution of publications according to the scientific scope of the journals is shown in Figure 1.

As shown in Figure 1, 63 (72.4%) of the publications were research articles, and 33 (37.9%) of all publications were in SCI/SCI-E journals in the first 10-year period. Although the second period covered only a 3.5-year timeframe, it is noteworthy that there was a considerable increase in publications compared to the first period (52.8% of the first 10-year period). This finding reflects an increased interest in pathology education in medical schools during the pandemic era. Especially, the experiences and various recommendations are shared on the use of methods such as distance learning and digital pathology.

**Figure 2:**
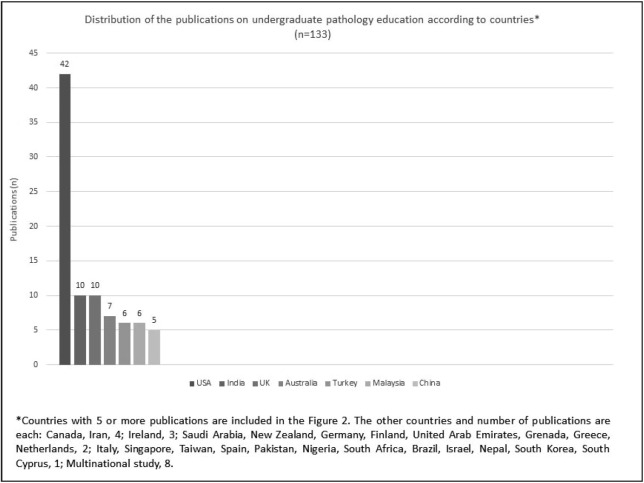
Distribution of the countries where studies on undergraduate pathology education were conducted between 2010-2023 June and the number of publications.

From 2010 to 2023 June, 66 different international journals, 19 of which (28.78%) were primarily focused on medical education, were published on the subject. The top 5 journals according to the frequency of the relevant publications were Arch Pathol Lab Med (SCI, 16 publications), Anat Sci Educ (SCI, 10 publications), Acad Pathol (ESCI, 9 publications), Human Pathol (SCI, 8 publications), and BMC Medical Education (SCIE, 8 publications). The top five countries publishing in this field were the United States of America (USA) (42 publications, 31.5%), India and the United Kingdom (10, 7.5%), Australia (7, 5.2%), and Turkey and Malaysia (6, 4.5%). Moreover, research was conducted in a total of 30 different countries from all continents, and 8 studies (6.0%) had a multinational setting. The distribution of the relevant publications according to the countries during the period between 2010 and 2023 June is shown in Figure 2.

## OPINIONS and EXPERIENCES REGARDING THE METHODS USED IN UNDERGRADUATE PATHOLOGY EDUCATION

### Views and Experiences in International Publications

#### 2010-2020 April

In the late 1980s, medical schools started to move towards system-based education. In this new curriculum, pathology, in conjunction with other basic and clinical disciplines, became part of the horizontal integrated education system. Accordingly, between 2010 and 2020 April, publications, especially reviews, emphasized the importance and benefits of the integrated system. During this period, another key topic was the recommendation for those responsible for education to be “facilitators” rather than "educators"; it was suggested that didactic lectures given by educators should be replaced by modern teaching methods that encourage students to learn on their own, such as flipped classrooms, problem/case-based learning, working in small groups, and peer teaching (1,5,6,8,9).

It is worth noting that in the period between 2010-2020 April, which reflects the period before the COVID-19 pandemic, articles on the use of digital microscopy in undergraduate medical education began to appear, albeit in small numbers. Ten articles published before 2020 compared digital microscopy and conventional light microscopy. In all of these studies, the benefits of pathology practices accompanied by digital pathology were emphasized (10–13).

During this period, articles about digital/web-based learning and distance education began to appear sporadically (6,11,14–18). Herbert et al. in 2017 and Onan et al. in 2019 discussed the blending of online and face-to-face education (blended learning) in their studies and described its benefits (6,19). In addition, new methods related to digital/web-based learning began to be developed and introduced. For example, research was published on using animations in distance education (15), digital game-based learning (16), enhancing learning with patient simulation software (17), and learning through online app platforms (14).

Various articles pointed out that autopsy education, which was popular in previous generations, was gradually decreasing, making it difficult for students to see macroscopic pathology and understand clinical-pathological correlations. Pathology museums, which were popular and widespread in the past, were disappearing worldwide, and there was a dramatic decrease in the number of autopsies (1). During the period between 2010 and 2020 April, six articles emphasized the importance of learning through autopsy, cadaver, and macroscopic specimens (20,21). However, the publications acknowledged the fact that access to autopsies or macroscopic specimens was becoming increasingly difficult. Fortunately, there are researchers seeking for solutions. For example, Nautiyal et al. suggested that macroscopic pathology education could be digital-based. The authors mentioned that digital-based images and videos could be used instead of surgical specimens fixed in formalin because there was no distortion caused by fixative, and specimens were better understood (11). Another solution was proposed by Mogali et al. who developed and presented high-quality macroscopic specimens marked with Quick Response (QR) codes (22). Another research group proposed the use of 3D-printed autopsy or macroscopic specimens (23).

In this period, some studies have been conducted to increase learning and analytical thinking. In some of these studies, students were made to prepare multiple-choice questions (24–26) or crossword puzzles (27) and it is reported that students’ analytical thinking skills improved, contributing to their learning.

**Figure 3:**
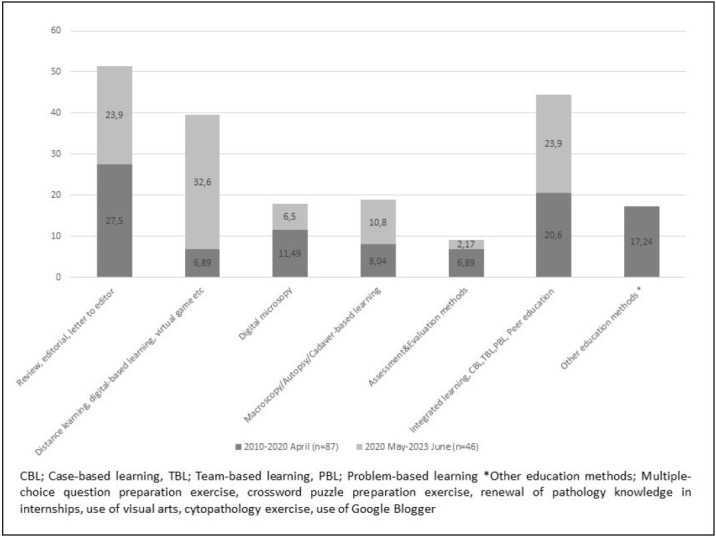
Distribution of the articles related to pathology education in medical schools from 2010 to 2023 June, according to topics (%)

Although measuring and evaluating the effectiveness of learning is a fundamental element of education, there are few publications on this topic. In the period from 2010 to 2020 April, six publications (6.89%) related to measurement and evaluation in pathology education in medical schools were identified (27–32). Patil and colleagues discussed the importance of balancing the distribution of topics and questions in exam preparation and the “blueprint” study they conducted for this purpose (30). The studies by Goneppanavar et al. and Htwe et al., shared experiences with objective structured practical examinations (OSPE) (27,28,31). Ho and colleagues discussed the benefits of their testing method using concept maps (32). In another study, it was mentioned that the practical examination in pathology microscopy and macroscopy, when conducted as a PowerPoint slide show on a computer, was more objective and structured compared to traditional desk-based exams, and saved time (31). Hu and colleagues questioned the relation between undergraduate and postgraduate exam success. In their study, students with high pathology performance in the pre-graduation period achieved higher scores in post-graduation exams such as United States Medical Licensing Examination (USMLE) and National Board of Medical Examiners (NBME) (29). Distribution of the articles according to the topics is shown in Figure 3.

#### 2020 May - 2023 June

There has been a significant change in the content of publications from 2020 May to June 2023, which also includes the period of COVID-19 pandemic restrictions. Among the published articles, 11 were in the form of reviews/editorials/letter to editorial, discussing the benefits and challenges of using distance learning and digital pathology for educational purposes, which gained in importance during the pandemic (8,33–37). Hassell and Afzal suggested encouraging and possibly mandating students in medical schools to use virtual pathology resources. It was mentioned that students taking elective virtual pathology internships would better understand the role of the pathologist in the healthcare team and the expectations from the pathology laboratory, and thus the number of doctors choosing pathology as their specialization could increase (33). Views on the pros and cons of virtual learning in pathology are summarized in Table 2.

**Table 2:** Positive and negative aspects of virtual learning in pathology.

**Possible Benefits**
Students have more autonomy. Students can choose where, when, how, and what to study on their own.
There are different curriculum options and modalities.
Short-segmented learning is possible from social media platforms like Facebook, Twitter, YouTube.
Performance and time spent can be tracked digitally .
Access to large number of institutions and a global community of educators is possible. Students can see different approaches to different situations.
Transportation, physical environment, and equipment costs are reduced.
A wider audience can participate in rarer events and training sessions.
Large databases can be used for educational purpose.s
**Disadvantages, Unexpected and Unintended Negative Consequences**
Technological issues on the educator or student side can reduce educational success.
Class participation may decrease. There is a possibility of dealing with other tasks during class.
Problems related to measurement and evaluation are higher.
Learning experiences that need to be acquired live (such as blood bank, microbiology, biochemistry) are insufficient.
Virtual learning environment is not suitable for kinesthetic (on-the job) learners.
Prolonged screen time and decreased physical activity can lead to vision, posture problems, as well as health problems like obesity, back pain etc.
Communication with patients, other doctors, and other team members is reduced and development of interpersonal communication skills might be insufficient.

Although the majority of research articles (n=18, 39.1%) came from the USA, articles have been published from 21 countries worldwide, including Turkey. In sixteen studies (34.78%), experiences related to distance education, digitally-based education, and digital microscopy have been shared (38–48). During this period, an increasing number of online education platforms have become available. In an article on this topic, it is explained that online platforms such as *PathPresenter* and *PathElective* can provide free, high-quality, organized pathology education to students. Besides, digital platforms like “*Discovering Pathology”* and “*Slice*” that gamify pathology education are mentioned (34).

After 2020, significant knowledge and experience were gained in the field of distance education. Furthermore, it became possible to compare experiences between distance and face-to-face education. Hernandez et al. reported success in student assessment and student satisfaction in remote pathology education during the pandemic, but emphasized that a hybrid education model might be more effective and beneficial (48). Tanaka and Ramachandran examined the benefits of remote education on both pathology residents and medical students, concluding that remote education was beneficial for students but emphasized that face-to-face education should not be abandoned for resident training (46). When students’ evaluations of distance education were considered, studies by Xu in China and Belezini in Greece found that medical students preferred traditional face-to-face education and believed that distance education should support it (47–49). In a study by Mastour in Iran, a study performed under a strict level of exam security, it was reported that significantly higher success rates were achieved in measurement and evaluation assessments compared to face-to-face education at the end of remote education (41).

In the post-2020 period, experiences with more interactive learning methods replacing didactic education have continued to be shared. Hassell and colleagues found it beneficial for educators to conduct small group work during pathology practical training, using annotated digital slides. They stated that integrated digital pathology case materials could be interactively used and incorporated into the education with flipped classroom, group work, and game formats, following a system-based curriculum (34,36). In twelve studies (26%), the contribution of modern methods such as integrated education and case-based/team-based/flipped classroom methods to learning has been examined (47,49–52). Studies evaluating case-based and team-based education in remote education during COVID-19, which included clinical information, laboratory tests, additional tests such as cytogenetics or flow cytometry, digital slides, radiological images, and macroscopic images, have reported positive results from both students and educators (34–38).

The studies comparing the effectiveness of digital pathology compared to light microscopy in pathology education in medical schools in the pre-COVID-19 period were replaced with reports on the use of telepathology in education during the pandemic (38,39). In all these publications, it was reported that digital pathology could be easily and successfully used in compulsory or elective pathology courses if the technical problems could be overcome (38,39). In a study conducted in Turkey, positive experiences with the use of telepathology in undergraduate pathology education during the COVID-19 period were reported (38). In this article, it was explained that the case-based learning experience was integrated into the distance education platform, in which the cases with clinical, radiological, and morphological features uploaded to the Virasoft Pathoclass system were presented and discussed by students in pathology practice courses during the pandemic. Student survey confirmed that various contemporary educational methods such as telepathology, case-based and team-based learning, and peer education were successfully used together during the practice courses (38).

In this period, there are four articles addressing the benefits of macroscopy and autopsy practices. In one of the studies emphasizing the importance of autopsy, students stated in questionnaires and open-ended questions that autopsy increased anatomical knowledge, observational skills, and clinical-pathological correlations (53). Another study suggested that autopsy not only helps students understand the pathophysiological mechanisms in disease processes but also enhances their experience in coping with the feeling of death (54). Suggestions have been made that this need can be met with virtual reality and augmented reality technologies. Augmented reality technology (*Google Glass, Alphabet Inc. and Microsoft HoloLens, Microsoft Inc.*) has been experimented within autopsy education, and it is promising although currently at an experimental level (34). Digitally enriched virtual autopsy cases on websites and similar digital platforms can fill the gap in autopsy education, and many such digital platforms can provide resources for both medical students and pathology assistants (34). According to Wan and colleagues, the *Interactive Digital Pathology Pool* they created using two- and three-dimensional high-resolution anatomical pathology images improved students’ success. They suggested that this pool could be enriched in the future by adding augmented reality and holographic images (55). Sutton-Butler and colleagues examined the effectiveness of the traditional method of examining macroscopic specimens in jars, a jar-based macroscopic specimen examination method, in an education study. In their study, 90% of students were satisfied with this education method, and 92% wanted this method to be used more frequently (56). Researchers suggested that returning to this traditional method could be beneficial for education, not only in anatomy and pathology but also in subjects such as ethics and organ donation (56).

In recent years, there has been an increasing number of articles in the literature about methods that make learning less monotonous and more enjoyable (57,58). In a study conducted in Egypt, the contributions of the Kahoot! game (57) and educational games played with flashcards, as mentioned in Schukow’s letter to the editor (59), were some examples of game-based learning. The use of social media platforms in education has also been studied, and Hamnvag and colleagues have highlighted the contributions of Twitter to learning (57–59).

Regarding measurement and evaluation methods in pathology, only one publication has been found in the literature for the period 2020 May to 2023 June. In this particular study, it was suggested to combine different types and weights of questions rather than a single type in order to evaluate the performance of the students in the most accurate way (48).

In our country and in the global literature, the number of studies examining the impact of pathology education provided in the preclinical years of a medical school on the clinical education years or in professional life is inadequate. Indeed, this knowledge will contribute to the rearrangement of the curriculum and teaching methods, and one of the few examples is the study by Hu et al. showing that success in medical school pathology courses correlated with post-graduation exam success (29). In our country, two studies have been conducted in this field. In the first one, a questionnaire-based survey was conducted with 5th-year medical students to inquire about the benefits of the pathology education they received during the preclinical period on their clinical years. Students considered case-based learning and macroscopy training as the most effective methods and suggested that they should be emphasized. It was reported that didactic lessons were more useful when combined with different methods such as case-based learning, assignments/projects, presentation, and microscopy studies (51). In the second study, a survey was conducted in a heterogeneous group of doctors from different specialties or general practitioners who graduated after 2000, with an objective to explore which method of undergraduate pathology education was most beneficial for their active professional lives. They stated that a functional and clinically integrated pathology curriculum and diversified modern learning methods provided the most significant benefit (50). These findings support approaches that have been increasingly emphasized since 2010, such as integrated curricula, case-based learning, the benefits of macroscopy, and the use of diversified modern educational techniques.

Although the number of publications on artificial intelligence (AI) applications in pathology have been increasing in recent years, publications focusing on the use of artificial intelligence in pathology education are limited. In a small number of recent reviews that describe the use of AI in pathology, possible options for its use in the education of medical students and pathology residents have been briefly mentioned (60–62). Models to assist residents in learning glomerulopathies (63) and cytopathologic materials (64) have been developed and tested. Evidently, AI algorithms that can help in areas such as assessment and evaluation, and curriculum development are needed in addition to supporting learning.

### Opinions and Experiences Published in Turkey

According to 2022 data, there are 118 medical schools and 143 programs in Turkey, with 97 of these schools being state-owned and 46 affiliated with private universities (65). A comprehensive study was published by Gençer and Dere in 2019, which is the most extensive study conducted in this field in Turkey and includes an analysis of the education programs of 41 state universities that provides medical education in Turkish and that use an integrated education system. According to this study, pathology courses begin in the second year in 58.2% (n=24) of medical schools, while they are offered in both the second and third years in 39%. In only one program (2.5%), pathology courses are included in the curriculum of the first year of medical school (66). In 7.3% of schools (n=3)), there is no pathology practice course. When all the schools included in the study are examined, the average theoretical course hours for pathology is 125.39 ± 28.11, and the average practical course hours is 26.21 ± 15.89. This study demonstrated that there is no standardization in the implementation, measurement, and evaluation of pathology education in our country. The authors drew attention to the significant differences in pathology education between schools in Turkey and emphasized the need for a standardized pathology education curriculum (66). A comprehensive analysis covering all state and private universities in Turkey to understand the general situation is currently not available.

In a study conducted regarding active physicians who graduated from medical schools in Turkey after 2000, 75% of the physicians (n=80) stated that pathology education was very useful or quite useful for their current professional lives, and 45% (n=48) found the education they received to be very adequate or quite adequate. The physicians considered the utility of the information (n=89, 83.2%), teaching methods (n=78, 72.9%), and the characteristics of the educator (n=75, 70.1%) to be important. They recommended case discussions (n=79, 72.9%), macroscopy (n=65, 60.7%), microscopy studies (n=62, 57.9%), problem-based learning (PBL) (n=61, 57.0%), and observation in the hospital laboratory (n=51, 47.6%) as major components of pathology education (51).

Sağol et al. shared their experiences with digital microscopy application in 2nd and 3rd-year medical students and reported that students easily adapted to the method, could review virtual slides from home, and had the opportunity to collaborate and participate interactively in classes (12). In a study conducted by Onan et al. before the COVID-19 pandemic with 3rd-grade students, an electronic education module and digital microscopy slides were added to traditional education. Students who used this blended teaching method in pathology education had higher success rates than those who did not. The authors reported that both students and educators were satisfied with the application (6). During the COVID-19 pandemic, telepathology began to be used in education in Turkey, as in the rest of the world, and it was applied by integrating it with case-based and team-based education (38,67) (Table 3).

**Table 3:** Publications on pathology education in medical schools in Turkey.

	**Authors /Year of Publication**	**Objective**	**Conclusion**
1	Sağol Ö, Yörükoğlu K, Lebe B, Durak MG, Ulukuş Ç, Tuna B, Musal B, Canda T, Özer E. / 2015 (12)	Investigation of the use of virtual microscopy in pathology practices and its impact on students and undergraduate education	The use of virtual microscopy was concluded to provide additional contribution to students’ pathology education.
2	Onan A, Usubütün A, Sezer B. / 2019 (6)	Investigation of the effect of a learner-centered and technology-supported teaching design in pathology education	In general, blended teaching design in pathology education increased student success and satisfaction. Both students and educators were satisfied with the implementation and wanted to continue to improve it.
3	Gençer CU, Dere Y. / 2019 (66)	To determine the place of pathology course in education programs of different medical schools	Standardizing and optimizing the capacity of faculty members and lecture hours will improve the quality of pathology education.
4	Şensu S, Teke Ö, Demirci M, Kutlu S, Genç BN, Ayaltı S, Gürbüz YS, Erdoğan N. / 2020 (38)	Transferring the experiences of distance education and telepathology during the COVID-19 pandemic	Telepathology combined with case-team based interactive learning can successfully be applied to education. It is important to integrate online learning into both theoretical and practical fields of medical education through the collaboration between health, computer, and education experts
5	Şensu S, Kutlu S, Gürbüz Y, Koçak H, Fışgın N, Erdoğan N. / 2022 (51)	Measurement and evaluation of the perceptions of 5th year medical students about the adequacy of the pathology education they received in the preclinical period and its benefit to their clinical education	Students consider case-based learning and macroscopy training as the most effective methods. It would be appropriate to combine didactic education with different and up-to-date learning methods and to update curriculum, accordingly.
6	Şensu S, Koçak H, Gürbüz Y, Fışgın N, Erdoğan N. / 2022 (50)	2000 and later medical graduates’ opinions and suggestions on the contribution of their undergraduate pathology education to their active professional lives, the adequacy and necessity of the education they received.	Pathology education will be useful in active professional life if the curriculum is functional, integrated with the clinic and provided with diversified up-to-date learning methods.

## CONCLUSION: QUESTIONS TO BE ANSWERED, SUGGESTIONS and WISHES

Notably, the publications in the last 10 years before the COVID-19 pandemic were focused on the weight of pathology within integrated education in medical schools and the transition from didactic education to active learning methods such as case /problem /team-based learning, flipped classrooms, and peer education was obvious. Additionally, digitalization started to find its place in educational methods. While these topics continued to be of interest after the COVID-19 pandemic, publications related to digital/web-based/remote education methods gained prominence. Various electronic learning platforms, some of them free and open to everyone, have been developed. The use of various digital games and social media platforms to facilitate and make learning enjoyable has become more widespread. However, experts continued to emphasize the importance of autopsies and macroscopy, recommending the use of virtual or augmented reality and 3D drawing applications to meet this need. It is worth noting that there are very few publications related to assessment and evaluation in pathology education in medical schools. It is an indisputable necessity to complete education by evaluating it with the most appropriate methods. Considering the use of artificial intelligence in pathology education is also a topic that needs consideration.

In Turkey, there is a need for comprehensive analyses that evaluate the infrastructure, curriculum, educational success, the number/quality of educators, and similar issues in undergraduate pathology education, covering all medical schools. Efforts should be made towards standardization in the implementation, measurement, and evaluation of pathology education. The following questions are awaiting answers: What should be the place and weight of pathology education in medical schools? What should be done to achieve learning objectives? How should learning outcomes be measured and evaluated? How can standardization be ensured and monitored? In a globalizing world, it should be kept in mind that not only national but also international and comprehensive standardization is necessary. In conclusion, it is believed that a joint effort is needed to achieve the desired goals in pathology education, and the answers to these questions can be found only with a collective intelligence.

## Conflict of Interest

The authors declare no conflict of interest.

## References

[ref-1] Buja L. Maximilian (2019). Medical education today: all that glitters is not gold. BMC Med Educ.

[ref-2] Yeoh Khay-Guan (2019). The future of medical education. Singapore Med J.

[ref-3] Khonglah Yookarin, Raphael Vandana, Mishra Jaya, Marbaniang Evarisalin, Chowdhury Zachariah, Dey Biswajit (2019). Relooking the curriculum and assessment in undergraduate pathology. J Educ Health Promot.

[ref-4] (uuuu). Mezuniyet Öncesi Tıp Eğitimi Ulusal Çekirdek Eğitim Programı 2020.

[ref-5] Osman Dr. Muhamed T, Kutty Dr. Methil Kannan (2012). Teaching of Pathology in a Hybrid Integrated Curriculum at a Malaysian Medical School. Int J Sci Res.

[ref-6] Onan Arif, Usubütün Alp, Sezer Barış (2019). Patoloji Eğitiminde Harmanlanmış Öğrenme Yaklaşımının Akademik Başarı ve Memnuniyet Üzerine Etkisi. Tıp Eğitimi Dünyası.

[ref-7] Sadofsky Moshe, Knollmann-Ritschel Barbara, Conran Richard M., Prystowsky Michael B. (2014). National standards in pathology education: developing competencies for integrated medical school curricula. Arch Pathol Lab Med.

[ref-8] Humphreys Hilary, Stevens Niall, Leddin Desmond, Callagy Grace, Burke Louise, Watson R. William, Toner Mary (2020). Pathology in Irish medical education. J Clin Pathol.

[ref-9] Grover Sumit, Sood Neena, Chaudhary Anurag (2017). Reforming pathology teaching in medical college by peer-assisted learning and student-oriented interest building activities: A pilot study. Educ Health (Abingdon).

[ref-10] Solberg Brooke L. (2012). Student perceptions of digital versus traditional slide use in undergraduate education. Clin Lab Sci.

[ref-11] Nautiyal Hemant K., Pathak Priyank, Nautiyal Ruchira, Sachdev Guruvansh, Pandey Shubham (2018). A comparative study of traditional and digital method in teaching surgical pathology to undergraduate medical students. Int Surg J.

[ref-12] Sağol Özgül, Yörükoğlu Kutsal, Lebe Banu, Durak Merih Güray, Ulukuş Çağnur, Tuna Burçin, Musal Berna, Canda Tülay, Özer Erdener (2015). Transition to Virtual Microscopy in Medical Undergraduate Pathology Education: First Experience of Turkey in Dokuz Eylül University Hospital. Turk Patoloji Derg.

[ref-13] Lee Bai-Chin, Hsieh Sung-Tsang, Chang Yih-Leong, Tseng Fen-Yu, Lin Yu-Jung, Chen Yuh-Lien, Wang Shu-Huei, Chang Yu-Fong, Ho Yi-Lwun, Ni Yen-Hsuan, Chang Shan-Chwen (2020). A Web-Based Virtual Microscopy Platform for Improving Academic Performance in Histology and Pathology Laboratory Courses: A Pilot Study. Anat Sci Educ.

[ref-14] Peacock Justin G., Grande Joseph P. (2016). An online app platform enhances collaborative medical student group learning and classroom management. Med Teach.

[ref-15] Sala Ripoll Cristina, Oparka Richard, Campbell Annie, Erolin Caroline (2017). The cell cycle: development of an eLearning animation. J Vis Commun Med.

[ref-16] Kanthan Rani, Senger Jenna-Lynn (2011). The impact of specially designed digital games-based learning in undergraduate pathology and medical education. Arch Pathol Lab Med.

[ref-17] Craig Fiona E., McGee James B., Mahoney John F., Roth Christine G. (2014). The Virtual Pathology Instructor: a medical student teaching tool developed using patient simulator software. Hum Pathol.

[ref-18] King Thomas S., Sharma Ramaswamy, Jackson Jeff, Fiebelkorn Kristin R. (2019). Clinical Case-Based Image Portfolios in Medical Histopathology. Anat Sci Educ.

[ref-19] Herbert Cristan, Velan Gary M., Pryor Wendy M., Kumar Rakesh K. (2017). A model for the use of blended learning in large group teaching sessions. BMC Med Educ.

[ref-20] Gopalan Vinod, Dissabandara Lakal, Nirthanan Selvanayagam, Forwood Mark R., Lam Alfred King-Yin (2016). Integrating gross pathology into teaching of undergraduate medical science students using human cadavers. Pathol Int.

[ref-21] Wood Andrew, Struthers Kate, Whiten Susan, Jackson David, Herrington C. Simon (2010). Introducing gross pathology to undergraduate medical students in the dissecting room. Anat Sci Educ.

[ref-22] Mogali Sreenivasulu Reddy, Vallabhajosyula Ranganath, Ng Chee Hon, Lim Darren, Ang Eng Tat, Abrahams Peter (2019). Scan and Learn: Quick Response Code Enabled Museum for Mobile Learning of Anatomy and Pathology. Anat Sci Educ.

[ref-23] Mahmoud Amr, Bennett Michael (2015). Introducing 3-Dimensional Printing of a Human Anatomic Pathology Specimen: Potential Benefits for Undergraduate and Postgraduate Education and Anatomic Pathology Practice. Arch Pathol Lab Med.

[ref-24] Herrero Jose Ignacio, Lucena Felipe, Quiroga Jorge (2019). Randomized study showing the benefit of medical study writing multiple choice questions on their learning. BMC Med Educ.

[ref-25] Olde Bekkink Marleen, Donders A. R. T. Rogier, Kooloos Jan G., Waal Rob Mw, Ruiter Dirk J. (2015). Challenging students to formulate written questions: a randomized controlled trial to assess learning effects. BMC Med Educ.

[ref-26] Grainger Rebecca, Dai Wei, Osborne Emma, Kenwright Diane (2018). Medical students create multiple-choice questions for learning in pathology education: a pilot study. BMC Med Educ.

[ref-27] Htwe T. T., Sabaridah I., Rajyaguru K. M., Mazidah A. M. (2012). Pathology crossword competition: an active and easy way of learning pathology in undergraduate medical education. Singapore Med J.

[ref-28] Goneppanavar Mangala, Dhaka Rajendra S. (2016). Undergraduate medical students' perspectives on objective structured practical examination in Pathology. Indian J Pathol Microbiol.

[ref-29] Hu Yinin, Martindale James R., LeGallo Robin D., White Casey B., McGahren Eugene D., Schroen Anneke T. (2016). Relationships between preclinical course grades and standardized exam performance. Adv Health Sci Educ Theory Pract.

[ref-30] Patil Sunita Y., Gosavi Manasi, Bannur Hema B., Ratnakar Ashwini (2015). Blueprinting in assessment: A tool to increase the validity of undergraduate written examinations in pathology. Int J Appl Basic Med Res.

[ref-31] Khan Sabina, Hassan Mohd Jaseem, Husain Musharraf, Jetley Sujata (2019). Video projected practical examination as an introduction to formative assessment tool for undergraduate examination in pathology. Indian J Pathol Microbiol.

[ref-32] Ho Veronica, Kumar Rakesh K., Velan Gary (2014). Online testable concept maps: benefits for learning about the pathogenesis of disease. Med Educ.

[ref-33] Hassell Lewis A., Afzal Anoshia (2021). Flattening the World of Pathology Education and Training and Shortening the Curve of Pathology Learning. Am J Clin Pathol.

[ref-34] Hassell Lewis A., Absar Syeda Fatima, Chauhan Chhavi, Dintzis Suzanne, Farver Carol F., Fathima Samreen, Glassy Eric F., Goldstein Jeffery A., Gullapalli Rama, Ho Jonhan, Koch Lisa K., Madory James E., Mirza Kamran M., Nguyen Phuong Nhat, Pantanowitz Liron, Parwani Anil, Rojansky Rebecca, Seifert Robert P., Singh Rajendra, ElGabry Ehab A., Bui Marilyn (2023). Pathology Education Powered by Virtual and Digital Transformation: Now and the Future. Arch Pathol Lab Med.

[ref-35] Ishak Angela, AlRawashdeh Mousa M., Meletiou-Mavrotheris Maria, Nikas Ilias P. (2022). Virtual Pathology Education in Medical Schools Worldwide during the COVID-19 Pandemic: Advantages, Challenges Faced, and Perspectives. Diagnostics (Basel).

[ref-36] Azimi Khatibani Seyed Esmaeil, Tabatabai Shima (2021). COVID-19 Impact on Modern Virtual Pathology Education: Challenges and Opportunities. Iran J Pathol.

[ref-37] Koch Lisa K., Chang Oliver H., Dintzis Suzanne M. (2021). Medical Education in Pathology: General Concepts and Strategies for Implementation. Arch Pathol Lab Med.

[ref-38] Şensu S, Teke O, Demirci M, Kutlu S, Genc BN, Ayalti S, Gurbuz YS, Erdogan N (2020). Telepathology in Medical Education: Integration of Digital Microscopy in Distance Pathology Education during COVID-19 Pandemic. JMSCR.

[ref-39] White Marissa J., Birkness Jacqueline E., Salimian Kevan J., Meiss Alice E., Butcher Monica, Davis Katelynn, Ware Alisha D., Zarella Mark D., Lecksell Kristen, Rooper Lisa M., Cimino-Mathews Ashley, VandenBussche Christopher J., Halushka Marc K., Thompson Elizabeth D. (2021). Continuing Undergraduate Pathology Medical Education in the Coronavirus Disease 2019 (COVID-19) Global Pandemic: The Johns Hopkins Virtual Surgical Pathology Clinical Elective. Arch Pathol Lab Med.

[ref-40] Rodrigues Maria Aparecida Marchesan, Zornoff Denise, Kobayasi Renata (2022). Remote Pathology teaching under the COVID-19 pandemic: Medical students' perceptions. Ann Diagn Pathol.

[ref-41] Mastour Haniye, Emadzadeh Ali, Hamidi Haji Abadi Omid, Niroumand Shabnam (2023). Are students performing the same in E-learning and In-person education? An introspective look at learning environments from an Iranian medical school standpoint. BMC Med Educ.

[ref-42] Manou Evangelia, Lazari Evgenia-Charikleia, Thomopoulou Georgia-Eleni, Agrogiannis Georgios, Kavantzas Nikolaos, Lazaris Andreas C. (2021). Participation and Interactivity in Synchronous E-Learning Pathology Course During the COVID-19 Pandemic. Adv Med Educ Pract.

[ref-43] Guiter Gerardo E., Sapia Sandra, Wright Alexander I., Hutchins Gordon G. A., Arayssi Thurayya (2021). Development of a Remote Online Collaborative Medical School Pathology Curriculum with Clinical Correlations, across Several International Sites, through the Covid-19 Pandemic. Med Sci Educ.

[ref-44] Hartsough Emily M., Arries Cade, Amin Khalid, Powell Deborah (2021). Designing and Implementing a Virtual Anatomic Pathology Elective During the COVID-19 Pandemic. Acad Pathol.

[ref-45] Cruz Myriam, Murphy Megan, Gentile Matthew M., Stewart Katherine, Barroeta Julieta E., Carrasco Gonzalo A., Kocher William D., Behling Kathryn C. (2021). Assessment of Pathology Learning Modules With Virtual Microscopy in a Preclinical Medical School Curriculum. Am J Clin Pathol.

[ref-46] Tanaka Kara S., Ramachandran Rageshree (2021). Perceptions of a Remote Learning Pathology Elective for Advanced Clinical Medical Students. Acad Pathol.

[ref-47] Xu Chun, Li Yiping, Chen Pingsheng, Pan Min, Bu Xiaodong (2020). A survey on the attitudes of Chinese medical students towards current pathology education. BMC Med Educ.

[ref-48] Hernandez Tahyna, Fallar Robert, Polydorides Alexandros D. (2021). Outcomes of Remote Pathology Instruction in Student Performance and Course Evaluation. Acad Pathol.

[ref-49] Belezini E, Katsoulas N, Thomopoulou GE, Lazaris AC (uuuu). The superiority of interactive courses combined with the teacher’s physical presence in the undergraduate pathology curriculum. Journal of Contemporary Medical Education.

[ref-50] Şensu S, Koçak H, Gürbüz YS, Taşdelen Fışgın N, Erdoğan N (2022). Hangi Yöntem Kullanılarak Yapılan Patoloji Eğitimi Aktif Meslek Hayatında Daha Yararlı? Klinisyenlerde Bir Anket Çalışması. Tıp Eğitimi Dünyası.

[ref-51] Şensu Sibel, Kutlu Sevcan, Gürbüz Yeşim Saliha, Koçak Hikmet, Taşdelen Fişgin Nuriye, Erdoğan Nusret (2022). Does the Pathology Education Received in the Undergraduate Preclinical Years Provide Benefits in the Clinical Education Period? A Survey on 5th Grade Medical Students. NKTD.

[ref-52] Cai Li, Li Yan-Li, Hu Xiang-Yang, Li Rong (2022). Implementation of flipped classroom combined with case-based learning: A promising and effective teaching modality in undergraduate pathology education. Medicine (Baltimore).

[ref-53] Hearle Patrick, Wong Wing Fei, Chan Joanna (2023). Undergraduate medical student perspectives on the role of autopsy in medical education. Acad Pathol.

[ref-54] Pakanen Lasse, Tikka Julius, Kuvaja Paula, Lunetta Philippe (2022). Autopsy-Based Learning is Essential But Underutilized in Medical Education: A Questionnaire Study. Anat Sci Educ.

[ref-55] Wan Ken Lee, Sen Arkendu, Selvaratnam Lakshmi, Naing Mohd Imran Mohd, Khoo Joon Joon, Rajadurai Pathmanathan (2022). Visual-spatial dimension integration in digital pathology education enhances anatomical pathology learning. BMC Med Educ.

[ref-56] Sutton-Butler Aoife, Croucher Karina, Garner Pip, Bielby-Clarke Keren, Farrow Matthew (2023). In jars: The integration of historical anatomical and pathological potted specimens in undergraduate education. Ann Anat.

[ref-57] Elkhamisy Fatma Alzahraa Abdelsalam, Wassef Rita Maher (2021). Innovating pathology learning via Kahoot! game-based tool: a quantitative study of students` perceptions and academic performance. Alexandria Journal of Medicine.

[ref-58] Hamnvåg Hans Magne, McHenry Austin, Ahmed Aadil, Trabzonlu Levent, Arnold Christina A., Mirza Kamran M. (2021). #TwitterHomework During Pathology Electives: Transforming Pathology Pedagogy. Arch Pathol Lab Med.

[ref-59] Schukow Casey P., Johnson Curtiss V., Kowalski Paul (2023). Is There Utility for Implementing Digital Flash Card Applications in Pathology Undergraduate and Graduate Medical Education?. Arch Pathol Lab Med.

[ref-60] Wells Amy, Patel Shaan, Lee Jason B., Motaparthi Kiran (2021). Artificial intelligence in dermatopathology: Diagnosis, education, and research. J Cutan Pathol.

[ref-61] Go Heounjeong (2022). Digital Pathology and Artificial Intelligence Applications in Pathology. Brain Tumor Res Treat.

[ref-62] Rakha Emad A., Toss Michael, Shiino Sho, Gamble Paul, Jaroensri Ronnachai, Mermel Craig H., Chen Po-Hsuan Cameron (2021). Current and future applications of artificial intelligence in pathology: a clinical perspective. J Clin Pathol.

[ref-63] Aldeman Nayze Lucena Sangreman, Sá Urtiga Aita Keylla Maria, Machado Vinícius Ponte, Mata Sousa Luiz Claudio Demes, Coelho Antonio Gilberto Borges, Silva Adalberto Socorro, Silva Mendes Ana Paula, Oliveira Neres Francisco Jair, Monte Semíramis Jamil Hadad (2021). Smartpathk: a platform for teaching glomerulopathies using machine learning. BMC Med Educ.

[ref-64] McAlpine Ewen, Michelow Pamela, Liebenberg Eric, Celik Turgay (2022). Is it real or not? Toward artificial intelligence-based realistic synthetic cytology image generation to augment teaching and quality assurance in pathology. J Am Soc Cytopathol.

[ref-65] Odabaşi Orhan (2023). Türkiye Tıp Fakülteleri 2023. STED.

[ref-66] Uğuz Gençer Ceren, Dere Yelda (2019). Pathology education in medical faculties. MAKU J Health Sci Inst.

[ref-67] Şensu S, Gürbüz Y, Ateş L, Erdoğan L, Hüseyinova I (2021). Case Based Learning Wıth Telepathology In Undergraduate Medical Education.

